# Predictive role of preoperative geriatric nutritional risk index for clinical outcomes in surgical gastric cancer patients: A meta-analysis

**DOI:** 10.3389/fsurg.2022.1020482

**Published:** 2022-11-02

**Authors:** Wei Lu, Jian Shen, Dehong Zou, Peng Li, Xiaocong Liu, Yi Jian

**Affiliations:** ^1^ Department of Gastroenterology, Chengdu Second People's Hospital, Chengdu, China; ^2^ Department of General Surgery, Chengdu Second People's Hospital, Chengdu, China

**Keywords:** geriatric nutritional risk index, clinical outcome, surgery, gastric cancer, meta-analysis, biomarker

## Abstract

**Purpose:**

The association between the preoperative Geriatric Nutritional Risk Index (GNRI) and postoperative short-term and long-term clinical outcomes remains unclear. The aim of this meta-analysis was to identify the predictive role of the preoperative GNRI for postoperative clinical outcomes of gastric cancer patients based on current evidence.

**Methods:**

Several databases were searched up to July 28, 2022. The primary and secondary outcomes were long-term survival, including overall survival (OS), cancer-specific survival (CSS) and postoperative complications. Meanwhile, the hazard ratios (HRs) and relative risks (RRs) with 95% confidence intervals (CIs) were combined to assess the association of preoperative GNRI with postoperative survival and complications separately. The results Eight studies involving 4,189 patients were included, and they were all from Japan. The pooled results demonstrated that a lower preoperative GNRI was significantly related to worse OS (HR = 1.72, 95% CI: 1.18–2.53, *P* = 0.005) and CSS (HR = 1.67, 95% CI: 1.20–2.32, *P* = 0.002). Meanwhile, a lower preoperative GNRI was significantly associated with postoperative complications (RR = 1.97, 95% CI: 1.51–2.58, *P* < 0.001). Further analysis focusing on elderly patients showed similar results.

**Conclusion:**

Preoperative GNRI is related to postoperative short-term and long-term clinical outcomes of Japanese gastric cancer patients, and a lower GNRI predicts poorer prognosis.

## Introduction

With the advances in medical technology and the improvement of the quality of life, the average life expectancy has obviously extended in the past few decades, especially in developed countries such as Japan, in which the proportion of the population aged ≥80 years has risen from 0.7% to 9.2% between 1960 and 2020 (1). Consequently, the proportion of elderly cancer patients has also increased significantly over the past few decades (2, 3). Gastric cancer is the sixth most common tumor worldwide, although the overall incidence of gastric cancer is expected to continue to decline (3). In addition, as life expectancy increases in some countries, its incidence in the elderly population also increases (4, 5).

Although the tumor-node-metastasis (TNM) staging system is currently the most authoritative tool for prognosis prediction, many indicators have been reported to play a role in predicting the short-term or long-term prognosis of gastric cancer patients, such as the neutrophil to platelet ratio (NPR) (6), red cell distribution width (RDW) (7), C-reactive protein to albumin ratio (CAR) (8), albumin to fibrinogen ratio (AFR) (9), prognostic nutritional index (PNI) (10), and systemic immune-inflammatory index (SII) (11). However, these parameters are not widely applied in clinics due to the disadvantages of instability and large differences in baseline values.

The nutritional status of the body is closely related to the progression and prognosis of cancer patients, and most gastric cancer patients are older (12). Thus, reliable indicators that can well reflect nutritional status and focus on elderly patients are considered to have high prognostic value for gastric cancer patients. The Geriatric Nutritional Risk Index (GNRI) is a novel and relatively reliable indicator that reflects the nutritional conditions of the body. It is calculated as 1.489 × serum albumin level (in g/l) + 41.7 × present weight/ideal weight (in kg), which indicates its stability to some extent. Furthermore, its prognostic role has been verified in several types of cancers, such as colorectal cancer, lung cancer and esophageal cancer, by meta-analyses (13–16). However, its predictive role in short-term and long-term prognosis in surgical gastric cancer remains unclear.

Thus, the aim of this meta-analysis was to identify the prognostic value of the preoperative GNRI for clinical outcomes in surgical cancer patients based on current evidence.

## Materials and methods

This meta-analysis was performed according to the Preferred Reporting Items for Systematic Reviews and Meta-Analysis (PRISMA 2009) checklist (17).

### Literature search

The PubMed, EMBASE and Web of Science electronic databases were searched from inception to July 28, 2022. The following terms were used during the search: geriatric nutritional risk index, GNRI, gastric, stomach, cancer, tumor, neoplasm, carcinoma, survival, prognostic, prognosis, surgery, operation, surgical and gastrectomy. The detailed search strategy was as follows: (geriatric nutritional risk index OR GNRI) AND (gastric OR stomach) AND (tumor OR cancer OR neoplasm OR carcinoma) AND (survival prognostic OR prognosis) AND (surgery OR operation OR surgical OR gastrectomy). Meanwhile, the references cited in the included studies were also reviewed for availability.

### Inclusion and exclusion criteria

The inclusion criteria were as follows: (1) patients were pathologically diagnosed with gastric cancer; (2) patients received surgical treatment, and GNRI was calculated as [1.489 × serum albumin level (in g/l)] + [41.7 × present weight/ideal weight (in kg)] before the surgery (14); (3) the association of preoperative GNRI with postoperative overall survival (OS), cancer-specific survival (CSS) or complications was explored; and (4) hazard ratios (HRs) with 95% confidence intervals (CIs) for OS and CSS were reported in the articles directly.

The exclusion criteria were as follows: (1) meeting abstracts, letters, editorials, animal trials or case reports; (2) low-quality studies with a Newcastle‒Ottawa Scale (NOS) score of 5 or lower (18); (3) duplicated or overlapped data; and (4) studies combining other malignancies.

### Data extraction

The following information was collected from the included studies: the name of the first author, publication year, country, sample size, age, tumor-node-metastasis (TNM) stage, cutoff value of GNRI, endpoint, NOS score, HR, RR and corresponding 95% CI.

### Methodological quality evaluation

Due to the retrospective nature of all included studies, the NOS score was applied to evaluate the methodological quality, and only studies with an NOS score of 6 or higher were included (18).

The literature search, selection, information collection and quality assessment were all conducted by two authors independently, and any disagreement was resolved by team discussion.

### Statistical analysis

In our meta-analysis, the HRs and RRs with corresponding 95% CIs were combined to explore the relationship between the preoperative GNRI and postoperative long-term survival and complications in gastric cancer patients. The heterogeneity between the included studies was calculated by *I*^2^ statistics and the Q test. When obvious heterogeneity was detected, presenting as *I*^2 ^> 50% or (and) *P *< 0.1, the random effects model was applied; otherwise, the fixed effects model was used (19). Sensitivity analysis was conducted to detect sources of heterogeneity and evaluate the impact of each included study on the overall results. All statistical analyses were conducted using STATA 12.0 software.

## Results

### Literature search and selection

**Figure 1:**
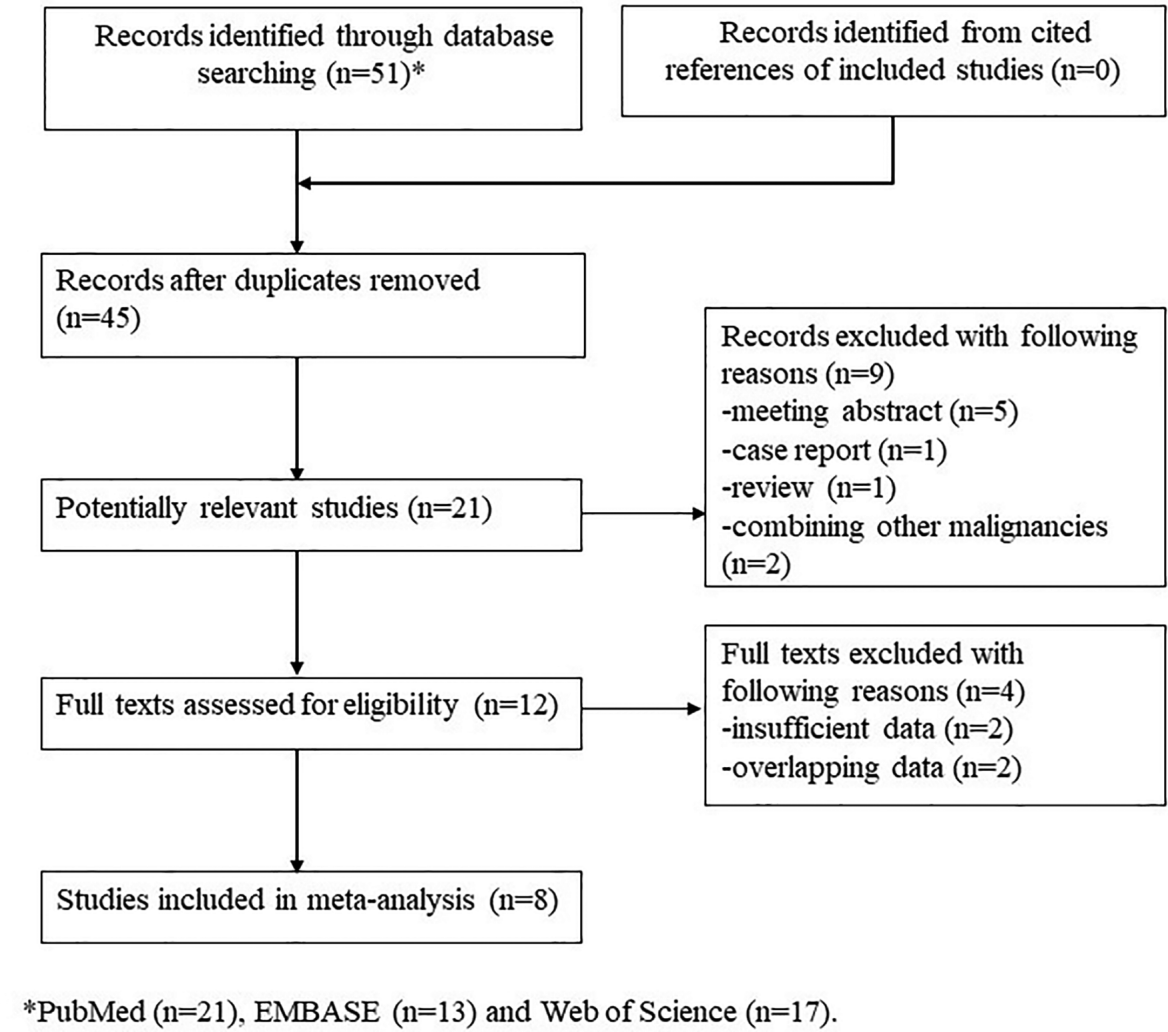
The flow diagram of this meta-analysis.

Initially, 51 records were found in three electronic databases, and 6 duplicated records were removed. After screening the titles, 24 irrelevant records were excluded, and 9 publications were further excluded after reviewing the abstracts. Finally, only eight retrospective studies were included after reviewing the full texts of the remaining publications (1, 20–26). The detailed process is presented in Figure 1.

### Basic characteristics of the included studies

All eight included studies were from Japan, and a total of 4,189 participants were enrolled. The sample size ranged from 234 to 1,166, and most included studies focused on elderly patients. Other specific information is shown in Table 1. Besides, as shown in Table 1, all included studies were high-quality studies with a NOS score of 6 or higher.

**Table 1:** Basic characteristics of included studies.

Author	Year	Country	Sample size	Age (year-old)	TNM stage	Cutoff value of GNRI	Endpoint	NOS
Kushiyama (20)	2018	Japan	348	>75	I-IV	92	PC	7
Furuke (21)	2021	Japan	795	68 (29–89)	I-III	92	OS, PC	8
Hirahara (22)	2021	Japan	303	≥65	I-III	85.7	OS, PC	7
Shimada (23)	2021	Japan	106	76 ± 7	NR	92	OS	6
Sugawara (24)	2021	Japan	1166	63 (25–91); 71 (28-91)	I-III	98	OS, CSS	7
Matsunaga (25)	2022	Japan	497	>75	I-III	97/95.8	OS, CSS	7
Toya (26)	2022	Japan	740	≥85	NR	NR	OS	6
Tsuchiya (1)	2022	Japan	234	≥80	I-III	98	OS, PC	7

NR, not reported; TNM, tumor-node-metastasis; GNRI, geriatric nutritional risk index; PC, postoperative complication; OS, overall survival; CSS, cancer-specific survival; NOS, Newcastle-Ottawa Scale.

### Results of the meta-analysis

**Figure 2:**
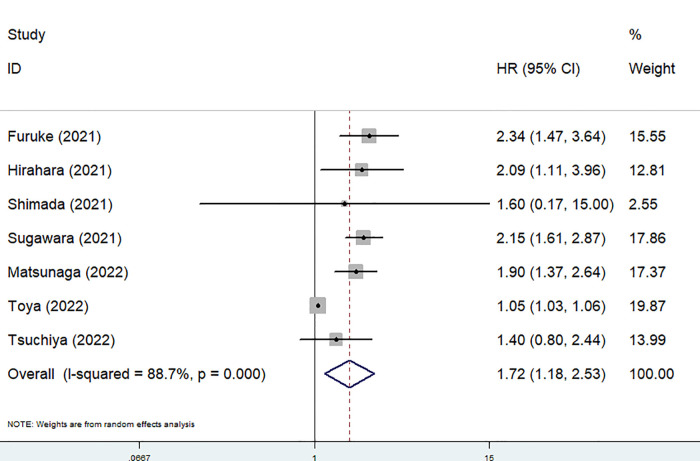
The association between preoperative geriatric nutritional risk index and overall survival of surgical gastric cancer patients.

Seven studies explored the relationship between the preoperative GNRI and OS in gastric cancer (1, 21–26). Due to the significant heterogeneity existed among these studies (*I*^2 ^= 88.7%, *P* < 0.001), also among studies focusing on elderly patients (*I*^2 ^= 78.0%, *P* = 0.001), the random effects model was applied. The pooled results showed that a lower preoperative GNRI was significantly associated with worse OS (HR = 1.72, 95% CI: 1.18–2.53, *P* = 0.005) (Figure 2), even focusing on elderly patients (HR = 1.49, 1.01–2.21, *P* = 0.046).

**Figure 3:**
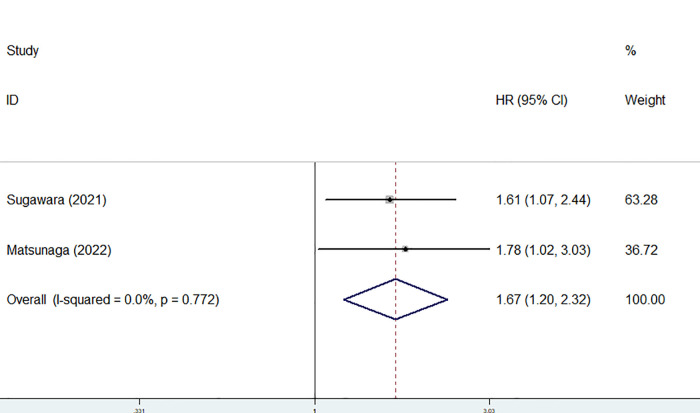
The association between preoperative geriatric nutritional risk index and cancer-specific survival of surgical gastric cancer patients.

Only two studies identified the association of the preoperative GNRI with the CSS of gastric cancer patients (24, 25) and there was no heterogeneity between the two studies (*I*^2 ^= 0.0%, *P* = 0.772). The pooled results showed a significant relationship between the preoperative GNRI and CSS of surgical gastric cancer patients (HR = 1.67, 95% CI: 1.20–2.32, *P* = 0.002) (Figure 3). The study by Matsunaga et al. focused on patients who were older than 75 years showed similar results (HR = 1.78, 95% CI: 1.03–3.03, *P* = 0.043).

**Figure 4:**
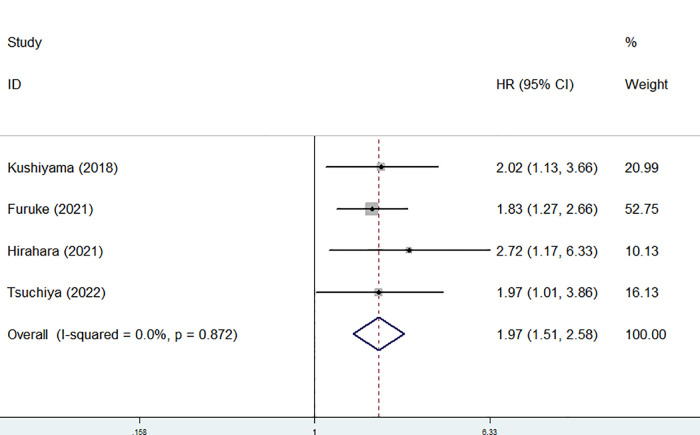
Sensitivity analysis for the association between preoperative geriatric nutritional risk index and overall survival of surgical gastric cancer patients.

In addition, the predictive role of the preoperative GNRI for postoperative complications in gastric cancer was explored in four studies (1, 20–22). None heterogeneity existed among these four studies (*I*^2 ^= 0.0%, *P* = 0.872), also among studies focusing on elderly patients (*I*^2 ^= 0.0%, *P* = 0.818). After combining these four studies, a lower preoperative GNRI predicted postoperative complications (RR = 1.97, 95% CI: 1.51–2.58, *P* < 0.001) (Figure 4), even focusing on elderly patients (HR = 2.13, 95% CI: 1.44–3.16, *P* < 0.001) (Table 2).

**Table 2:** Results of meta-analysis.

	No. of studies	HR/RR	95% CI	*P* value	*I*^2^ (%)	P_heterogeneity_
Overall survival	7 (1, 21–26)	1.72	1.18–2.53	0.005	88.7	< 0.001
Elderly patients	5 (1, 22, 23, 25, 26)	1.49	1.01–2.21	0.046	78.0	0.001
Cancer-specific survival	2 (24, 25)	1.67	1.20–2.32	0.002	0.0	0.772
Elderly patients	1 (25)	1.78	1.03–3.03	0.043	-	-
Postoperative complication	4 (1, 20–22)	1.97	1.51–2.58	< 0.001	0.0	0.872
Elderly patients	3 (1, 20, 22)	2.13	1.44–3.16	< 0.001	0.0	0.818

HR, hazard ratio; RR, relative risk; CI, confidence interval.

### Sensitivity analysis and publication bias

**Figure 5:**
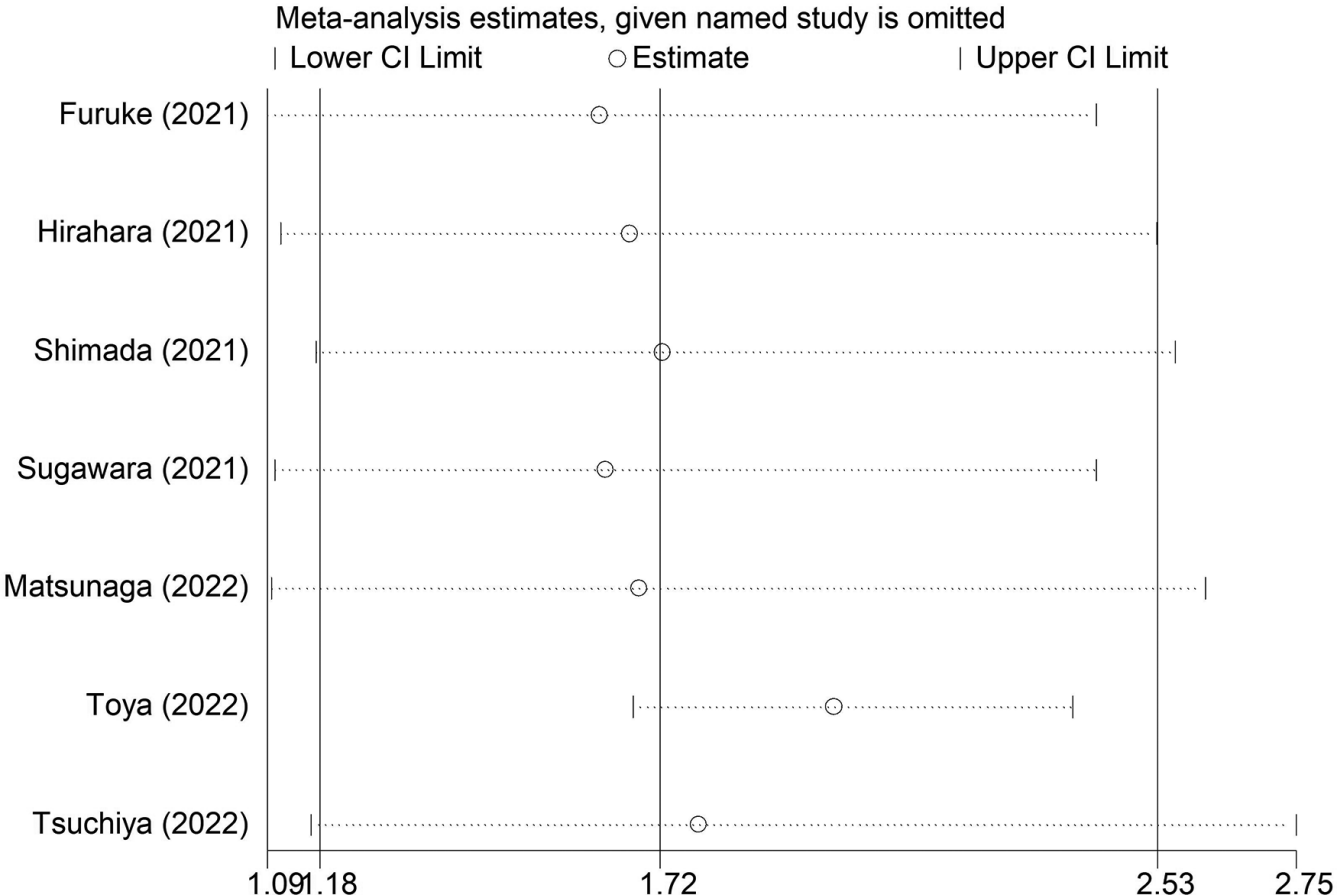
Begg's funnel plot.

The sensitivity analysis indicated that the overall results for OS were stable and reliable, and none of the included studies had a significant impact on the overall results (Figure 5).

## Discussion

The current meta-analysis suggested that a lower preoperative GNRI predicted postoperative complications and worse long-term survival in Japanese gastric cancer patients, even in elderly patients. The GNRI might serve as a promising and reliable prognostic indicator and help to develop appropriate treatment strategies. However, more prospective high-quality studies from other countries are still needed to further validate our findings.

To date, multiple meta-analyses have explored the prognostic role of the GNRI in cancer patients. Zhao et al. included nine studies involving 3,658 participants and demonstrated that colorectal cancer patients with a lower baseline GNRI showed worse OS (HR = 2.39, 95% CI: 1.78–3.23, *P* < 0.001) and progression-free survival (PFS) (HR = 1.77, 95% CI: 1.38–2.26, *P* < 0.001) (14). Sun et al. showed that a lower GNRI was significantly related to poor OS (HR = 2.01, 95% CI: 1.65–2.44, *P* < 0.001) and PFS/CSS (HR = 1.81, 95% CI: 1.48–2.22, *P* < 0.001) (16). Furthermore, their results also indicated that a low GNRI was associated with histological type [odds ratio (OR) = 1.55, 95% CI: 1.19–2.03, *P* = 0.001] and Eastern Cooperative Oncology Group performance status (ECOG PS) (OR = 2.81, 95% CI: 1.49–5.32, *P* = 0.001) (16). In addition, Yu et al. verified that a lower pretreatment GNRI was related to poorer OS (HR = 1.47, 95% CI: 1.33–1.63, *P* < 0.001) and PFS (HR = 1.69, 95% CI: 1.24–2.31, *P* = 0.001) (27). Based on our results, the preoperative GNRI is considered to be a reliable prognostic factor for surgical gastric cancer patients.

For gastric cancer, malnutrition is one of the most common comorbidities and risk factors for poor prognosis due to the physiological function of the stomach. Malnutrition leads to immunosuppression, which has been identified as a risk factor for poor prognosis in gastric cancer patients (28). Immunosuppression usually causes poor responses to some antitumor therapies, such as chemotherapy, and promotes tumor progression and recurrence (29). Moreover, malnutrition is closely related to postoperative complications, and postoperative complications induce systemic inflammation, which is associated with immunosuppression and poor prognosis in gastric cancer patients (29–32). Thus, preoperative malnutrition leads to poor clinical outcomes after surgery in gastric cancer.

Several nutritional indexes have been introduced and reported to play a role in predicting the prognosis of gastric cancer patients. The controlling nutritional status (CONUT) score is calculated from the serum albumin level, total cholesterol level and total lymphocyte count and has been demonstrated to be associated with the incidence of postoperative complications and poor survival in the meta-analysis by Takagi et al. (33). PNI is calculated from serum albumin level and total lymphocyte count, and its prognostic value in surgical gastric cancer has been well verified in the meta-analysis conducted by Li et al. after including 25 studies (34). However, these indicators are easily affected by some conditions, such as inflammatory diseases. The ratio of present weight/ideal weight is applied in the calculation of GNRI. Thus, it is believed that the GNRI might be more stable and reliable and play an important role in predicting the clinical outcomes of gastric cancer patients.

Actually, there are several fields about the GNRI in gastric cancer that need further investigation. First, the value of the GNRI might change during antitumor treatment, which causes damage to the nutritional status of the body in some cases, and the dynamic changes in the GNRI during therapy are believed to show some prognostic value. Second, as mentioned above, nutritional condition is an important factor affecting the prognosis of gastric cancer patients. Thus, we deem that increasing the GNRI may help improve the prognosis, which should be identified by more studies. Third, a great number of gastric cancer patients suffer from nutrition-related complications after surgery, such as dumping syndrome and hypoglycemia syndrome. Thus, the postoperative GNRI might also play a role in predicting the prognosis of surgical gastric cancer patients.

There are several limitations in this meta-analysis. First, all included studies were retrospective, and the overall sample size was relatively small, which might cause some bias. Second, all included studies were from Japan, which limits the generalizability of our findings. Third, we were unable to conduct more detailed analyses due to the lack of original data. Fourth, in this type of research, it is difficult to further determine the optimal cutoff value of the preoperative GNRI. Five, significant heterogeneity for OS was observed in our meta-analysis, and the sources of heterogeneity remain unclear.

## Conclusion

The preoperative GNRI is significantly associated with postoperative short-term and long-term clinical outcomes in Japanese gastric cancer patients, and a lower GNRI predicts poorer prognosis. However, more prospective high-quality studies from other countries are still needed to further verify our findings.

## Data Availability

The original contributions presented in the study are included in the article/Supplementary Material, further inquiries can be directed to the corresponding author/s.
